# No Evidence for Disease History as a Risk Factor for Narcolepsy after A(H1N1)pdm09 Vaccination

**DOI:** 10.1371/journal.pone.0154296

**Published:** 2016-04-27

**Authors:** Favelle Lamb, Alexander Ploner, Katharina Fink, Markus Maeurer, Peter Bergman, Fredrik Piehl, Daniel Weibel, Pär Sparén, Lisen Arnheim Dahlström

**Affiliations:** 1 Department of Medical Epidemiology and Biostatistics, Karolinska Institutet, Stockholm, Sweden; 2 Department of Clinical Neuroscience, Karolinska Institutet, Stockholm, Sweden; 3 CAST (Centre for allogenic stem cell transplantation), Karolinska Hospital, Stockholm, Sweden; 4 Division of Therapeutic Immunology (TIM), LabMed Karolinska Institutet, Stockholm, Sweden; 5 Division of Clinical Microbiology, Department of Laboratory Medicine, Karolinska Institutet and Karolinska University Hospital, Huddinge, Stockholm, Sweden; 6 Department of Medical Informatics, Erasmus University Medical Center, Rotterdam, The Netherlands; Oasi Institute for Research and Prevention of Mental Retardation, ITALY

## Abstract

**Objectives:**

To investigate disease history before A(H1N1)pdm09 vaccination as a risk factor for narcolepsy.

**Methods:**

Case-control study in Sweden. Cases included persons referred for a Multiple Sleep Latency Test between 2009 and 2010, identified through diagnostic sleep centres and confirmed through independent review of medical charts. Controls, selected from the total population register, were matched to cases on age, gender, MSLT-referral date and county of residence. Disease history (prescriptions and diagnoses) and vaccination history was collected through telephone interviews and population-based healthcare registers. Conditional logistic regression was used to investigate disease history before A(H1N1)pdm09 vaccination as a risk-factor for narcolepsy.

**Results:**

In total, 72 narcolepsy cases and 251 controls were included (range 3–69 years mean19-years). Risk of narcolepsy was increased in individuals with a disease history of nervous system disorders (OR range = 3.6–8.8) and mental and behavioural disorders (OR = 3.8, 95% CI 1.6–8.8) before referral. In a second analysis of vaccinated individuals only, nearly all initial associations were no longer statistically significant and effect sizes were smaller (OR range = 1.3–2.6). A significant effect for antibiotics (OR = 0.4, 95% CI 0.2–0.8) and a marginally significant effect for nervous system disorders was observed. In a third case-only analysis, comparing cases referred before vaccination to those referred after; prescriptions for nervous system disorders (OR = 26.0 95% CI 4.0–170.2) and ADHD (OR = 35.3 95% CI 3.4–369.9) were statistically significant during the vaccination period, suggesting initial associations were due to confounding by indication.

**Conclusion:**

The findings of this study do not support disease history before A(H1N1)pdm09 vaccination as a risk factor for narcolepsy.

## Introduction

Between October 2009 and March 2010 during the A(H1N1)pdm09 influenza pandemic, vaccination with AS03-adujvanted A(H1N1)pdm09 vaccine was carried out in Sweden with an estimated coverage of 60% [1]. Following the vaccination campaign, several cases of narcolepsy were reported to the Swedish Medical Products Agency, leading to concern over a possible association between the vaccine and narcolepsy. This notion was subsequently confirmed in a registry study in children under 19-years [2]. Similar associations between vaccination and narcolepsy have been reported in several studies from other countries [3–7]. It is still unclear if this association is linked to the AS03 adjuvant used in the A(H1N1)pdm09 vaccine or particular features of the specific influenza antigen component [8, 9].

In 2014, the International Classification of Sleep Disorders (ICSD-3) introduced two new terms for the classification of narcolepsy: Type-1 narcolepsy and Type-2 narcolepsy [10]. Prior to this point, narcolepsy was known as either narcolepsy with cataplexy or narcolepsy without cataplexy [11]. Narcolepsy has been shown to be strongly associated with the HLA-DQB1*06:02 allele and the loss of hypothalamic hypocretin (orexin)-producing neurons results in the development of narcolepsy [12–14]. This has led to the speculation that narcolepsy may be an immune mediated disease [12, 15]. Additional findings from genetic studies support the possibility that vaccinations could act as environmental triggers for narcolepsy [12, 16–18]. In addition to underlying genetic susceptibility, epidemiological and seroepidemiological studies have looked at the role of chronic diseases [12, 19, 20], streptococcal infection as a trigger [21, 22] and that infection with H1N1 could itself play a role in the development of narcolepsy [22–25]. However the role of disease history, e.g. chronic diseases, cancer, respiratory diseases, bacterial diseases, viral diseases and mental/behavioural disorders for the association between A(H1N1)pdm09 vaccine and narcolepsy has rarely been studied. With an increasing body of evidence now linking narcolepsy with an autoimmune reaction and potentially streptococcal and H1N1 infection, it is therefore relevant to study disease history, especially autoimmune manifestations and infections, to determine any evidence of such an association.

## Methods

### Study setting

This was a nationwide retrospective case-control study with a source population of approximately 9.4 million people between 2009 and 2010 [26]. It is built upon original methodology as part of the Vaccine Adverse Event Surveillance and Communication (VAESCO), European Centre of Disease Control [27].

### Case and control identification

Cases were identified through diagnostic sleep centres and/or neurophysiology labs in Sweden, with the six largest centres (out of seven nationwide) participating. The centres provided a list of potential cases i.e. a list of individuals referred for a Multiple Sleep Latency Test (MSLT) during the study period, 1^st^ January 2009 and 31^st^ December 2010. Of the 431 potential cases, 142 received a primary diagnosis of narcolepsy by the initial referring clinician. Karolinska Institutet contacted these preliminarily diagnosed cases to request their participation in the study.

**Fig 1:**
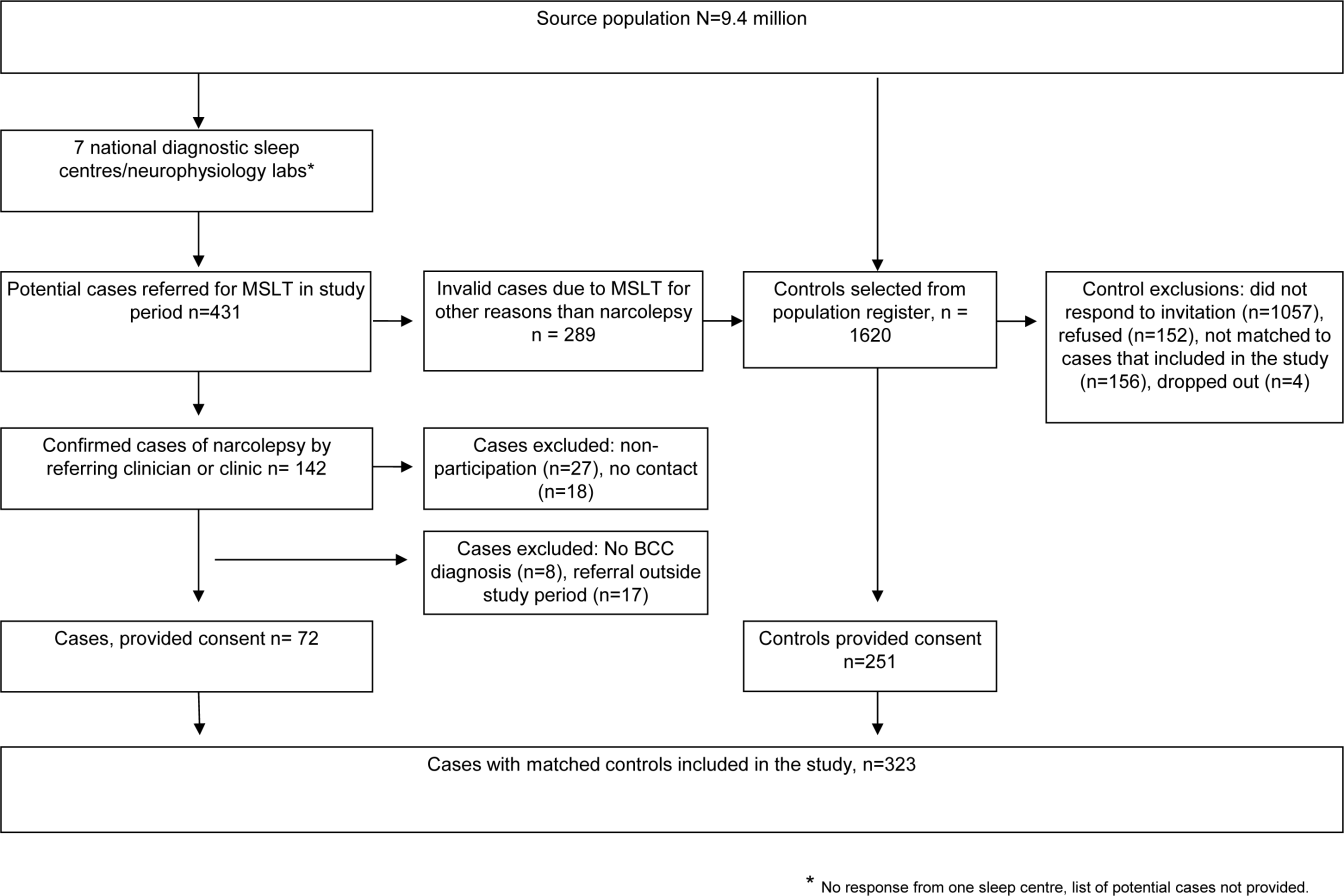
Flowchart to show case and control identification.

Of the 142 preliminary diagnosed cases, 27 refused participation and 18 were not contactable. The medical charts of the remaining 97 subjects were reviewed by a neurologist blinded to A(H1N1)pdm09 vaccination status. Consequently, 17 subjects were excluded as they were discovered to have an MSLT referral date outside the study period and eight did not have a diagnosis of narcolepsy according to the Brighton Classification Criteria [28]. This left, a total of 72 confirmed cases for inclusion in the study (Fig 1). The date when a case was first referred for a MSLT was defined as the index date.

Controls were selected to match each case on age, gender, index date (so that the control did not have an MSLT-referral or narcolepsy diagnosis before its matched case), and county of residence. These were randomly selected from the Swedish Total Population Register, held by Statistics Sweden [29]. Four matched controls per case were invited to participate in the study. Of these, 251 individuals accepted the invitation (1057 did not respond, 152 refused participation, a further 156 were excluded, as they were not matched to cases that agreed to be part of the study, and four subsequently dropped out). (Fig 1).

### Ethical approval

This study was approved by the regional ethics committee in Stockholm and written informed consent was collected from all study participants.

### Data collection

Information regarding MSLT referral date and diagnosis date was collected from medical charts for the cases. Additional information for onset of cataplexy, excessive daytime sleepiness and Hypocretin-1 measurement in CSF was also available, however due to incomplete/missing/unknown dates for some cases these variables were excluded from the analysis.

Using a standardised questionnaire, a research nurse collected self-reported information, via telephone interviews, on previous disease and vaccination history from cases and controls for the period 1^st^ January 2005 - 31^st^ December 2010. Additional information on methodology can be found in the ECDC technical report [27]. Due to this long ascertainment period, information on self-reported medications and diseases was often incomplete i.e. the answer to simple yes/no questions regarding use of medication and diseases was missing in up to 45% of respondents, and the corresponding dates of use/disease were missing for between 73% and 100% (data not shown).

Consequently, self-reported information on diseases and medications was discarded in favour of register data, with the exception of vaccination status, which was only available through interview as the registration of vaccinations was not mandatory during the study period. Information on medication use was collected from the Prescribed Drug Register (PDR), which includes data on drugs prescribed in Sweden from July 2005 including prescription date, dosage and duration, expenditure and reimbursements. All drugs are classified according to the World Health Organisation Anatomical Therapeutic Chemical (ATC) Classification [30]. Disease information was collected from the National Patient Registers (NPR), which contains information on diagnoses in Swedish hospitals from specialised outpatient care from 2001 and inpatient care since 1987. The registers are managed by the National Board of Health and Welfare [31] and linkage of participant data to the registers is possible through their unique personal identification numbers [32].

### Exposures

Data on the following ATC codes were collected to investigate whether prescription history was a risk factor for narcolepsy before A(H1N1)pdm09 vaccination: nervous system disorders (N), obstructive airway diseases (R03), antibacterial (J01), anti-inflammatory/anti-rheumatic drugs, non-steroid (M01A), antiviral (J05), immunosuppressant (L04) and diabetes (A10). Prescriptions for psychostimulants, agents used for ADHD treatment and nootropics (ATC N06BA01, N06BA03, N06BA04, N06BA07, and N06BA09) were used as a proxy for ADHD diagnosis in this study. The ADHD subcategory was created from prescriptions for N and therefore prescriptions for ADHD are included in the total number of prescriptions for N. Prescription issue date is used as a proxy for date of drug use.

Data on the following diagnosis codes, based on the International Classification of Disease (ICD) version 10, were collected from the NPR to investigate whether diagnosis history was a risk factor for narcolepsy before A(H1N1)pdm09 vaccination: nervous system disorders (G00-473, G475-99 –excluding narcolepsy (G474)), mental and behavioural disorders (F00-99), bacterial diseases (A30-49), neoplasms (C00-99), respiratory diseases (J00-99), viral infections of the CNS (A80-99), other infections of CNS (B25-34), other bacterial, viral and other infectious agents (B95-99) and diabetes mellitus (E10-14).

To investigate if a combination of prescription (ATC) and disease (ICD10) history was a risk factor for narcolepsy before A(H1N1)pdm09 vaccination, four combined exposure groups were created: a) nervous system disorders (ATC N and/or ICD10 G00-473, 475–99); b) bacterial diseases (ATC J01/ ICD10 A30-49); c) respiratory diseases (ATC R03/ICD10 J00-99) and; d) viral diseases (ATC J05/ ICD10 A80-99, B25-34).

For the purpose of measuring a crude measure of intensity a multiple prescription/diagnosis variable was created. This variable took into account that participants may have received one or more prescriptions and/or diagnoses over the exposure period in more than one prescription or diagnosis category.

### Exposure windows

**Fig 2:**
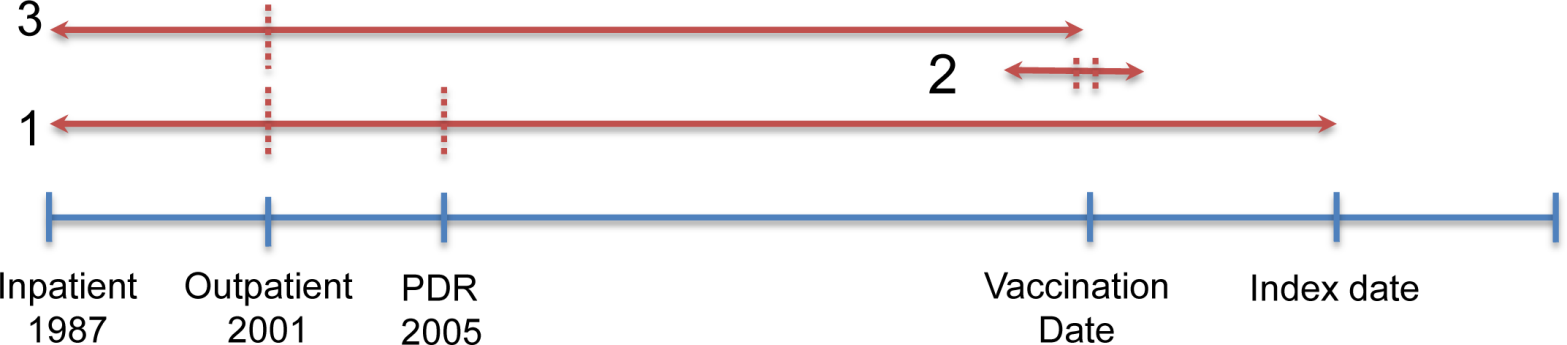
Exposure windows. 1. Prescription (ATC) and diagnosis (ICD10) history before index date (MSLT-referral date). Ever was defined as after the first date in either the inpatient register (1987), outpatient register (2001) or prescribed drug register (2005) and prior to the index date. 2. Prescription (ATC) and diagnosis (ICD10) history during the vaccination period. This period extended to six months before to one month after A(H1N1)pdm09 vaccination (specific to each individual). For acute infections (bacterial (ATC J01) and viral (ATC J05)) this period was shortened to two weeks before to two weeks after vaccination. 3. Prescription (ATC) and diagnosis (ICD10) history before vaccination. Ever was defined as after the first date in either the PDR (2005), inpatient register (1987) or outpatient register (2001) and prior to the vaccination date.

Disease history was studied as a risk factor for narcolepsy over three different exposure windows (Fig 2): 1) prescription (ATC) and diagnosis (ICD10) history before the index date. This period covered at least 5-years prior to the index date from the first prescription in the PDR (2005), or diagnosis date in the inpatient (1987) or outpatient (2001) registers respectively; 2) prescription history during the ‘vaccination period’ defined as six months before each case/control was first vaccinated with A(H1N1)pdm09 vaccine until one month after, with the exception of acute infections, e.g. prescriptions for antibacterials (ATC J01) and antivirals (ATC J05), where this period was shortened to two weeks before and after, as these infections are self-limiting; 3) prescription and diagnosis history before A(H1N1)pdm09 vaccination—again covering at least 5-years prior to vaccination, from first prescription or diagnosis.

The self-reported A(H1N1)pdm09 vaccination dates were incomplete for some cases and controls. In such instances the day of month was assigned the 15^th^, missing months in 2009 were assigned to November i.e. the middle of October-December, and missing months in 2010 were assigned to January i.e. the middle of the overall vaccination period of October 2009-March 2010.

### Statistical analysis

Conditional logistic regression was used to estimate odds ratios (OR) together with 95% confidence intervals (95% CI). Given the rarity of narcolepsy in the general population, ORs were interpreted as the relative risk (RR) of narcolepsy [33]. Each exposure was assessed as a covariate in a univariate model, which was controlled for the joint effect of the matching variables through conditioning. The significance level was 0.05 and all tests were two sided. The analyses were performed using SAS version 9.4.

Three analyses were performed. First, a full data analysis investigating disease history as a risk factor for narcolepsy. In this model, all cases and controls were included regardless of vaccination status and timing of vaccination, i.e. whether the index date was before A(H1N1)pdm09 vaccination or after. The exposure window of interest in this analysis was prescriptions or diagnoses before the index date (exposure window 1, Fig 2).

Second, a vaccinated-only analysis i.e. cases and controls vaccinated before the index date, to determine if disease history is an effect modifier for vaccination status. The periods of interest were prescriptions and diagnosis history before the index date (exposure window 1), prescription history during the vaccination period (exposure window 2) and prescription and diagnosis history before vaccination (exposure window 3, Fig 2).

Third, a case-only analysis was used to compare vaccinated cases with an index date before vaccination to vaccinated cases with an index date after vaccination. This analysis is based on the assumption that for a rare disease, with somewhat unspecific symptoms, there may be an extended history of misdiagnoses/treatment prior to the correct diagnosis, leading to confounding by indication. The exposure windows of interest in this analysis included prescription and diagnosis history during the vaccination period and before vaccination (exposure window 2 and 3 respectively, Fig 2).

Sensitivity analyses using the diagnosis date of narcolepsy as the index date instead of MSLT-referral date were carried out for all three analyses.

## Results

The majority of cases (71%) were under 20 years of age and a larger proportion of cases were vaccinated compared to the controls (94% vs. 74%) (Table 1).

**Table 1:** Characteristics of the study population.

Characteristics	Case (N = 72)	Control (N = 251)
	N (%)	N (%)
Sex (male)	35 (48.6)	124 (49.4)
H1N1 vaccination	68 (94.4)	186 (74.1)
Age (years)		
<5	4 (5.6)	18 (6.1)
6–10	13 (18.1)	49 (19.5)
11–15	17 (23.6)	60 (23.9)
16–20	17 (23.6)	51 (20.3)
21–30	9 (12.5)	32 (10.9)
30+	12 (16.7)	41 (14.0)

In the full data analysis several prescription and diagnosis categories before the index date were associated with an increased risk of narcolepsy (Table 2 and exposure window 1, Fig 2). Statistically significant effects were found for prescriptions of drugs for nervous system disorders (OR = 3.6, 95% CI 1.8–7.1), prescriptions for ADHD (OR = 4.5, 95% CI 1.4–14.7), diagnosis of nervous system disorders (OR = 8.8, 95% CI = 2.7–28.6) and diagnoses of mental and behavioural disorders (OR = 3.8, 95% CI 1.6–8.8), Further statistically significant findings were found for prescriptions for obstructive airway diseases, multiple prescriptions and multiple disease diagnoses and combined prescriptions/diagnoses for nervous system disorders (Table 2).

**Table 2:** Full case-analysis for prescription (ATC) history and diagnosis (ICD10) history before index date and vaccinated-only analysis for prescription and diagnosis history before index date on the risk of developing narcolepsy.

Characteristics	Full-case analysis*				Vaccinated-only analysis**			
	Cases	Controls	OR (95% CI)	P-value	Cases	Controls	OR (95% CI)	P-value
	(N = 72)	(N = 251)			N = 54)	(N = 85)		
	N (%)	N (%)			(N (%)	N (%)		
**Prescriptions (ATC) before index date**								
Nervous system disorders (N)	21 (29.17)	30 (11.95)	3.55 (1.77–7.13)	**0.0004**	15 (27.78)	19 (22.35)	1.34 (0.61–2.93)	0.47
ADHD (N06BA)	7 (9.72)	5 (1.99)	4.49 (1.37–14.71)	**0.01**	6 (11.11)	4 (4.71)	2.56 (0.68–9.42)	0.17
Obstructive airway diseases (R03)	13 (18.05)	31 (12.35)	2.14 (1.00–4.57)	**0.049**	11 (20.37)	15 (17.65)	1.19 (0.50–2.84)	0.69
Antibacterial (J01)	43 (59.72)	127 (50.60)	1.58 (0.90–2.77)	0.12	32 (59.26)	67 (78.82)	0.39 (0.18–0.83)	**0.01**
Anti-inflammatory/rheumatic drugs, non-steroid (M01A)	7 (9.72)	30 (11.95)	0.84 (0.33–2.14)	0.71	5 (9.26)	15 (17.65)	0.48 (0.16–1.40)	0.18
Antiviral (J05)	2 (2.78)	6 (2.39)	1.23 (0.24–6.23)	0.80	2 (3.70)	3 (3.53)	1.05 (0.17–6.51)	0.96
Multiple prescriptions	57 (79.17)	142 (56.57)	3.30 (1.72–6.33)	**0.0003**	43 (79.63)	73 (85.88)	0.64 (0.26–1.58)	0.34
**Diagnoses (ICD10) before index date**								
Nervous system disorders (G00-473, 475–99)	10 (13.89)	6 (2.40)	8.76 (2.68–28.61)	**0.0003**	7 (12.96)	3 (3.53)	4.07 (1.01–16.50)	**0.049**
Mental and behavioural disorders (F00-99)	13 (18.06)	14 (5.58)	3.76 (1.60–8.81)	**0.002**	10 (18.52)	7 (8.24)	2.53 (0.90–7.12)	0.08
Bacterial diseases (A30-49)	1 (1.39)	1 (0.40)	7.48 (0.47–119.82)	0.15	1 (1.85)	1 (1.18)	1.59 (0.10–25.88)	0.75
Neoplasms (C00-99)	1 (1.39)	3 (1.20)	1.36 (0.12–15.98)	0.81	1 (1.85)	1 (1.18)	1.59 (0.10–25.88)	0.75
Respiratory disease (J00-99)	15 (20.83)	58 (23.12)	0.98 (0.48–1.99)	0.96	12 (22.22)	27 (31.76)	0.61 (0.28–1.35)	0.22
Multiple diagnoses	31 (43.06)	71 (28.29)	2.39 (1.30–4.39)	**0.005**	26 (48.15)	34 (40.0)	1.39 (0.70–2.77)	0.35
**Prescriptions (ATC) &/or diagnoses (ICD10) before index date**								
Nervous system disorders (N/G00-473, 475–99)	27 (37.50)	33 (13.15)	3.96 (2.17–7.23)	**<0.0001**	20 (37.04)	21 (24.71)	1.79 (0.86–3.76)	0.12
Bacterial disease (J01/A30-49)	43 (59.72)	126 (50.20)	1.47 (0.86–2.50)	0.16	32 (59.26)	67 (78.82)	0.39 (0.18–3.76)	0.12
Respiratory diseases (R03/J00-99)	22 (37.50)	67 (26.69)	1.21 (0.68–2.15)	0.52	19 (35.19)	31 (36.47)	0.95 (0.46–1.93)	0.88
Viral diseases (J05/A80-99, B25-34)	2 (2.78)	7 (2.79)	0.10 (0.20–4.90)	0.10	2 (3.70)	4 (4.71)	0.78 (0.14–4.41)	0.78

*Full case-model includes all cases and controls, regardless of vaccination status and timing of vaccination.

** Case exclusions in vaccinated-only analysis: 4 unvaccinated, 2 missing vaccination date, 1 missing referral date, 10 referred before vaccination, 1 ambiguous vaccination date. Control exclusions: 64 unvaccinated, 79 missing vaccination dates, 3 matched to case with no referral date, 20 matched with cases referred before vaccination. Prescription (ATC) and diagnoses (ICD10) categories investigated but insufficient data to compute from model: Prescriptions before index date; immunosuppressant (L04) and diabetes (A10). Diagnoses before index date; viral infections of CNS (A80-99), other viral infections of CNS (B25-34), other bacterial, viral and other infectious agents (B95-99) and diabetes mellitus (E10-14).

In the second, vaccinated-only analysis, nearly all statistically significant associations from the full data analysis were no longer significant (Table 2). Only diagnoses of nervous system disorders before index date (exposure window 1, Fig 2) appeared marginally significant (OR = 4.1 95% CI 1.01–16.5); this is not only due to lower power and wider confidence intervals, but we also found a consistent reduction of the estimated relative risks. In addition, it was found that the prescriptions of drugs for antibiotics before index date had a significant protective effect (OR = 0.4, 95% CI 0.2–0.8).

Furthermore, in the second vaccinated-only analysis, no statistically significant associations were found for prescriptions or diagnoses during the vaccination period (exposure window 2, Fig 2) or diagnoses or multiple prescription/diagnoses before vaccination (exposure window 3, Fig 2). The only statistically significant effect was observed for prescriptions of drugs for nervous system disorders before vaccination, which was protective (OR = 0.3 95% CI 0.1–1.0) (Table 3).

**Table 3:** Vaccinated-only analysis investigating prescription (ATC) history during the vaccination period and ever before A(H1N1)pdm09 vaccination and diagnosis (ICD10) history ever before vaccination and prior to index on the risk of developing narcolepsy.

Characteristics	Cases*	Controls**	Odds ratio (95% CI)	P-value
	N = 54 (%)	N = 85 (%)	Unadjusted	
**Prescriptions (ATC) in vaccination period**				
Nervous system disorders (N)	2 (3.70)	8 (9.41)	0.37 (0.08–1.81)	0.22
ADHD (N06BA)	1 (1.85)	4 (4.71)	0.38 (0.04–3.51)	0.40
Obstructive airway disease (R03)	3 (5.56)	5 (5.88)	0.94 (0.22–4.11)	0.94
Anti-inflammatory/rheumatic drugs, non-steroid (M01A)	1 (1.85)	4 (4.71)	0.52 (0.05–5.09)	0.57
Multiple prescriptions	6 (11.11)	14 (16.47)	0.63 (0.23–1.77)	0.38
**Prescriptions (ATC) before vaccination**				
Nervous system disorders (N)	3 (5.56)	15 (17.65)	0.28 (0.08–1.0)	**0.049**
ADHD (N06BA)	1 (1.85)	3 (3.53)	0.52 (0.05–5.09)	0.57
Obstructive airway diseases (R03)	10 (18.52)	14 (16.47)	1.15 (0.47–2.82)	0.76
Antibacterial (J01)	30 (55.56)	58 (68.24)	0.58 (0.29–1.18)	0.13
Antiviral (J05)	1 (1.85)	3 (3.53)	0.52 (0.05–5.09)	0.57
Anti-inflammatory/rheumatic drugs, non-steroid (M01A)	5 (9.26)	13 (15.29)	0.57 (0.19–1.69)	0.31
Multiple prescriptions	35 (64.81)	66 (77.65)	0.53 (0.25–1.13)	0.10
**Diagnoses (ICD10) before vaccination**				
Nervous system disorders (G00-473, 475–99)	1 (1.85)	3 (3.53)	0.52 (0.05–5.09)	0.57
Mental behavioural disorders (F00-99)	4 (4.71)	7 (8.24)	0.89 (0.25–3.20)	0.86
Respiratory disease (J00-99)	11 (20.37)	26 (30.59)	0.58 (0.26–1.30)	0.19
Bacterial disease (A30-49)	1 (1.85)	1 (81.12)	1.59 (0.10–25.88)	0.75
Multiple diagnoses	18 (33.33)	33 (38.82)	0.79 (0.39–1.61)	0.51
**Prescriptions (ATC) &/or diagnoses (ICD10) before vaccination**				
Nervous system disorders (N/ G00-473, 475–99)	4 (7.41)	17 (20.0)	0.32 (0.10–1.01)	0.052
Bacterial disease (J01/A30-49)	30 (55.56)	58 (68.24)	0.58 (0.29–1.12)	0.13
Respiratory diseases (R03/J00-99)	17 (31.48)	29 (34.12)	0.89 (0.43–1.84)	0.75
Viral disease (J05/A80-99, B25-34)	1 (1.85)	4 (4.71)	0.38 (0.04–3.51)	0.40

*Case exclusions: 4 unvaccinated, 2 missing vaccination date, 1 missing referral date, 10 referred before vaccination, 1 ambiguous vaccination date.

**Control exclusions: 64 unvaccinated, 79 missing vaccination dates, 3 matched to cases with no referral date, 20 matched with cases referred before vaccination. Prescription (ATC) and diagnoses (ICD10) categories investigated but insufficient data to compute from the model: Prescriptions in the vaccination period; antibacterial (J01), antiviral (J05), immunosuppressant (L04) and diabetes (A10). Prescriptions before index date; immunosuppressant (L04) and diabetes (A10). Diagnoses before vaccination; neoplasms (C00-99), viral infections of CNS (A80-99), other infections of CNS (B25-34), other bacterial, viral and other infectious agents (B95-99), and diabetes mellitus (E10-14).

The third and final case-only analysis compared vaccinated cases with an index date before vaccination to vaccinated cases with an index date after vaccination (Table 4): Large significant effects were observed for prescriptions of drugs for nervous system disorders (OR = 26.0, 95% CI 4.0–170.2), ADHD (OR = 35.3, 95% CI 3.4–369.9) and anti-inflammatory/rheumatic drugs (OR = 13.3, 95% CI 1.1–163.5) during the vaccination period (exposure window 2, Fig 2). Multiple prescriptions were also statistically significant (Table 4).

**Table 4:** Case-only analysis investigating prescription (ATC) history during the vaccination period and before index date and diagnosis (ICD10) history before index date on risk of developing narcolepsy.

Case characteristics	Referral before vaccination	Referral after vaccination	Odds ratio (95% CI)	P-value
N = 64*	N = 10 (%)	N = 54 (%)	Unadjusted	
**Prescriptions (ATC) in vaccination period**				
Nervous system disorders (N)	5 (50.0)	2 (3.70)	26.00 (3.97–170.21)	**0.0007**
ADHD (N06BA)	4 (40.0)	1 (1.85)	35.33 (3.38–369.85)	**0.003**
Obstructive airway diseases (R03)	1 (10.0)	3 (5.56)	1.89 (0.18–20.24)	0.60
Anti-inflammatory/rheumatic drugs, non-steroid (M01A)	2 (20.0)	1 (1.85)	13.25 (1.07–163.52)	**0.04**
Multiple prescriptions	5 (50.0)	6 (11.11)	8.00 (1.78–35.94)	**0.007**
**Prescriptions (ATC) before vaccination**				
Nervous system disorders (N)	5 (50.0)	3 (5.56)	17.00 (3.10–93.12)	**0.001**
ADHD (N06BA)	5 (50.0)	1 (1.85)	53.00 (5.13–547.26)	**0.0009**
Obstructive airway diseases (R03)	1 (10.0)	10 (18.52)	0.49 (0.06–4.31)	0.52
Anti-inflammatory/rheumatic drugs, non-steroid (M01A)	2 (20.0)	5 (9.26)	2.45 (0.40–14.85)	0.33
Antibacterial (J01)	6 (60.0)	30 (55.56)	1.20 (0.30–4.74)	0.79
Multiple prescriptions	7 (70.0)	35 (64.81)	1.27 (0.29–5.47)	0.75
**Diagnoses (ICD10) before vaccination**				
Nervous system disorders (G00-473, 475–99)	3 (30.0)	1 (1.85)	0.04 (0.004–0.48)	**0.01**
Mental and behavioural disorders (F00-99)	3 (30.0)	4 (7.71)	0.19 (0.03–1.02)	0.052
Respiratory diseases (J00-99)	1 (10.0)	11 (20.37)	2.30 (0.26–20.15)	0.45
Multiple diagnoses	4 (40.0)	18 (33.34)	0.75 (0.19–3.00)	0.68
**Prescriptions (ATC) &/or diagnoses (ICD10) before vaccination**				
a) Nervous system disorders (N/ G00-473, 475–99)	6 (60.0)	4 (7.71)	18.75 (3.70–95.14)	**0.0004**
b) Bacterial diseases (J01/A30-49)	6 (60.0)	30 (55.56)	1.20 (0.30–4.74)	0.79
c) Respiratory diseases (R03/J00-99)	1 (10.0)	17 (31.48)	0.24 (0.03–2.06)	0.19

*Excluding 4 unvaccinated cases, 2 cases missing vaccination date, 1 case no referral date, 1 case with ambiguous vaccination date.

Prescription (ATC) and diagnoses (ICD10) categories investigated but insufficient data to compute from the model: Prescriptions during vaccination period; antibacterial (J01) antiviral (J05), immunosuppressant (L04) and diabetes (A10), Prescriptions (ATC) before index date; antiviral (J05), immunosuppressant (L04) and diabetes (A10). Diagnoses before index date; bacterial diseases (A30-49), neoplasms (C00-99), viral infections of CNS (A80-99), other infections of CNS (B25-34), other bacterial, viral and other infectious agents (B95-99), and diabetes mellitus (E10-14). Prescriptions &/or diagnoses before index date; viral disease (J05/A80-99, B25-34).

Large significant effects were found for prescriptions for nervous system disorders (OR = 17.0 95% CI 3.1–93.1) and ADHD (OR = 53.0 95% CI 5.1–547.3) before vaccination (Exposure window 3, Fig 2). A statistically significant effect was observed for diagnoses of nervous system disorders before vaccination, which was protective (OR = 0.04 95% CI 0.0004–0.48) (Table 4).

Using the diagnosis date as the index date rather than MSLT-referral date the same patterns of statistical significance in the sensitivity analysis were found (data not shown).

## Discussion

Three analyses of the risk of narcolepsy based on vaccination status and prior disease history were undertaken using data from a nationwide population-based case-control study. No evidence was found that prior disease history represented a risk factor for narcolepsy and it is probable that statistically significant associations found in the initial full data analysis are likely a result of confounding by indication.

In the first full data analysis, disease history before the index date (exposure window 1) was investigated as a risk factor for narcolepsy. An increased risk of narcolepsy for prescriptions of drugs for nervous system disorders, ADHD and obstructive airway diseases and prior diagnosis of nervous system disorders and mental and behavioural disorders was found. It is hypothesised that the significant effect observed for ADHD could be explained by potential misdiagnosis, which could then be a contributory factor for the statistically significant effects observed for prescriptions for nervous system disorders and diagnoses of mental and behavioural disorders. When comparing the overall neurological/psychiatric disorders between cases and controls in our study, only cases were previously diagnosed with sleep related diseases such as hypersomnia and sleep apnoea, which would support the idea that early cases of narcolepsy may have been initially misdiagnosed (data not shown).

Oosterloo et al [34] have showed that there is a risk of misdiagnosis between hypersomnias of central origin, e.g. narcolepsy and ADHD in adults, since both share symptoms such as problems with concentration and attention. Furthermore, Modestino et al [35] reported that adults with narcolepsy often displayed ADHD symptomatology in childhood, supporting the possibility that early cases in our study may have been initially misdiagnosed and prescribed ADHD medications. There is also the potential for overlap in treatment between ADHD and narcolepsy, with some medications such as Adderall® (amphetamine, dextroamphetamine mixed salts) being used to treat both conditions [36]. Together, such factors offer a potential explanation for the statistically significant effect observed for the prescription of drugs for ADHD in the full data analysis.

While a growing number of studies have looked at streptococcal infection and H1N1 infection as a risk factor for narcolepsy [21–25], we found no evidence for such an association in our study. Due to the small study size, it was not possible to look at individual disease codes in the broader ATC & ICD10 categories, resulting in a risk of type II statistical error due to lack of power. In addition, we based our analysis on health care register data, which may have resulted in a registration of fewer bacterial and viral infections being reported, with people only seeking medical care when an infection is severe and longer lasting. This may have resulted in an underestimation of disease exposure and be a potential reason for the lack of associations found here.

A second vaccinated-only analysis was carried out excluding cases or controls that were unvaccinated or had an index date (MSLT-referral) prior to vaccination. Interestingly, nearly all statistically significant associations from the full data analysis were no longer significant and there was a consistent reduction of the estimated relative risks.

As this second analysis had excluded cases with an index date before vaccination (early cases), it was speculated that such cases could be driving the associations observed in the full data analysis: if early cases presented with symptoms before vaccination then they most probably would have received more prescriptions within the vaccination period (and before index date) than those referred after vaccination (vaccine-associated cases). Additionally, the diagnostic time frame is shorter in vaccine-associated cases relative to naturally occurring cases, which may have resulted in fewer incorrect prescriptions and diagnoses in cases with an index date after vaccination. In addition, due to an increased awareness of narcolepsy following A(H1N1)pdm09 vaccination, this too could have resulted in less misdiagnosis of vaccine-associated cases. Such factors could help to explain how early cases might be driving the statistically significant effects observed in the full data analysis and further indicate that exposure window 1, i.e. disease history before index date, could be contaminated with early misdiagnosis between the administration of the vaccination and index date.

In order to establish whether there was a difference in disease history between early cases and cases and vaccine-associated cases, a third analysis was conducted. It was found that early cases were far more likely to have received prescriptions of drugs for nervous system disorders, ADHD, anti-inflammatory/rheumatic drugs and multiple prescriptions before and during the vaccination period. This suggests that the statistically significant effects from the full data analysis were most likely due to confounding by indication and that disease history before A(H1N1)pdm09 vaccination is not a risk factor for narcolepsy.

### Strengths and limitations

To reduce the effects of confounding, strict definitions were used for inclusion criteria and we matched cases on age, gender, index date and county of residence. At the time of case ascertainment, Type-1 and Type-2 narcolepsy definitions had not been introduced. Due to incomplete/missing dates for symptom onset e.g. cataplexy, EDS and hypocretin-1 measurement for some cases, old case definitions were kept. This is a limitation of the study. However, it is unlikely that the lack of this information would have changed the results, nor would it have helped us answer whether previous disease history was a risk factor for narcolepsy. To compensate for missing dates and reduce recall bias in the self-reported information, we used exposure information from the Swedish healthcare registers. The A(H1N1)pdm09 vaccination date was self-reported however. Although we controlled for self-reported vaccination dates we cannot rule out the possibility of recall bias, with cases being more likely to remember if and when they received their vaccination than controls, resulting in an overestimation of exposure in cases. However, as this study took place shortly after the vaccination campaign we believe this reduced the potential effects of recall bias. To account for the time between vaccination and diagnosis, several exposure windows were analysed specific to each individual and based on/around the date they first received the A(H1N1)pdm09 vaccine, or their index date.

Diagnostic bias was a potential issue in this study as typically, many narcolepsy cases have an incremental progression of symptoms over several years, which results in a lag between the onset of symptoms and diagnosis. However, following the vaccination campaign there was an increased awareness of narcolepsy, which shortened the time between the onset of symptoms and diagnosis. To reduce the potential for diagnostic bias in this study, the MSLT-referral date was used as the index date and not the date of diagnosis.

It is of note that the ATC and ICD codes used in this study are only proxies for a number of diseases and they have broad underlying aetiologies, so while they can provide estimations for disease history they are not as specific as individual codes. In addition, people are less likely to seek medical attention for milder infections, which could have resulted in an underestimation of such exposures in this study. It was unfortunately not possible to look at individual codes in this study, due to a limited sample size. While lack of power is a limitation in this study, it still remains one of the larger observational studies to date in Sweden, which looks at the association between A(H1N1)pdm09 vaccination and narcolepsy.

## Conclusions

The findings from this study do not support that disease history is a risk factor for narcolepsy after A(H1N1)pdm09 vaccination. However, due to the limited power of this study these results require to be confirmed through larger studies to investigate the role of disease history on the aetiology of narcolepsy. Additional studies are required to explore the possible misdiagnosis of narcolepsy in childhood with ADHD and other mental and behavioural disorders, to determine whether narcolepsy cases are being missed until later years.
